# Responding to A Hazardous Materials Incident in Nepal

**DOI:** 10.31729/jnma.9029

**Published:** 2025-05-31

**Authors:** Naveen Phuyal, Ashis Shrestha

**Affiliations:** 1 Department of Community Medicine, Nepalese Army Institute of Health Sciences, Sanobharyang, Kathmandu, Nepal; 2 Department of Emergency Medicine, Patan Academy of Health Sciences, Lagankhel, Lalitpur, Nepal

**Keywords:** *hazarduous materials*, *HAZMAT*, *Nepal*

## Abstract

Paper talks about hazardous materials incidents in Nepal that threaten health, safety and the environment. Rapid urbanisation and industrial growth have made chemical, biological, radiological and nuclear emergencies more likely, but there is still not a clear national plan or complete chemical inventory, leaving the health sector unprepared. Emergency responders rely on the Emergency Response Guidebook and the Wireless Information System for Emergency Responders, but many are not aware of these tools or trained to use them. The Nepalese Army has a dedicated chemical, biological, radiological and nuclear platoon, yet hospitals still lack decontamination protocols, equipment and trained staff. Co-ordination between agencies is weak, resources are limited and exercises are rare. It feels like the pieces do not fit together. We suggest developing national guidelines aligned with international standards, forming dedicated response teams, running regular training sessions and including chemical incident plans in hospital disaster plans to improve preparedness.

## HAZMAT AND HAZMAT INCIDENT

Hazardous materials (HAZMAT) are substances or materials that pose potential risks to health, safety, or the environment due to their chemical, physical, or biological properties. These include chemicals, gases, radioactive materials, biological agents, and more. Hazardous materials may cause harm through exposure, inhalation, ingestion, or physical contact, and they are regulated to prevent accidents, pollution, and health risks.^1^

Hazardous materials can be found everywhere in the community, like factories, hospitals, and even homes as well as transported by water, land, and air transport. If released, they have the potential to cause harm to people, property, critical infrastructures, and the environment. There are a wide range of people who can get affected by HAZMAT incidents: people who handle hazardous material daily, transportation carriers, people residing near industrial areas, first responders, and first receivers. There is a wide range of health risks associated with Hazardous Materials which are broadly classified as Thermal, Radiological, Asphyxiation, Chemical, Etiological (Biological), and Mechanical (TRACEM).^2^

A HAZMAT incident refers to an occurrence where hazardous substances, which may be chemical, biological, radiological, or nuclear (CBRN), are accidentally or intentionally released, posing a potential risk to human health, safety, property, or the environment. These incidents can happen during the handling, transportation, storage, or disposal of hazardous materials and often require immediate and specialized response measures to contain, mitigate, and prevent further harm.^3^ This article seeks to identify the current situation of HAZMAT incident management in Nepal, collate available information related to HAZMAT incident management internationally, and provide suggestions appropriate for Nepal's context.

## THE SITUATION OF HAZMAT MANAGEMENT IN NEPAL

HAZMAT incident is a concern for a country like Nepal, because of its rapid urbanization, and industrial growth but limited infrastructure for the management of HAZMAT incidents. Nepal still does not have a chemical profile, although the Environment Protection Act 2053 states that all industries that produce toxic chemical waste must declare them in the Nepal Gazette. Although no official confirmation of any major chemical events has been documented in Nepal, the risk remains high due to the usage of hazardous and toxic chemicals in the textile industries and agriculture sectors. Nepal is a member of the Strategic Approach to International Chemical Management (SAICM) policy and does have the Acid and Other Dangerous Chemical Substance Regulation Act, 2080, these policies and acts however, do not cover the management of a major chemical incident. Although the Nepal Army has a dedicated CBRN platoon, Nepal does not have a national chemical strategy, nor does it have a public health plan for managing chemical incidents and emergencies at the site. The hospitals in Nepal have Hospital Disaster Preparedness and Response Plans (HDPRP), which has a section on managing outbreaks but managing chemical incidents is still lacking in the plan. In addition, no dedicated health management centers exist to cater to mass chemical incidents.^4^

## HAZMAT IDENTIFICATION AND MANAGEMENT RESOURCES

HAZMAT incident management is a complex activity, so to simplify the task and ensure clear decisionmaking, the first responders use a risk-based process for response called 'APEI' which stands for 'Analyze, Plan, Implement and Evaluate.'^5^ Regarding tactical management of hazardous material emergencies at the incident site an eight-step process is recommended as a flexible guideline. The eight step process starts with site management and control, the establishment of an incident command post, establishing staging area, isolation perimeter, zoning of the perimeter as a hot, warm, and cold zone, and ends with the termination of the incident response.^6^

Access to HAZMAT training and resources is limited for a Low Middle-Income country like Nepal. However, first responders may be able to access such information and identify and respond to HAZMAT incidents by using resources available on the internet. If the first responders want to rapidly identify and respond to the hazardous material involved in transportation-related accidents, the Emergency Response Guidebook (ERG) is a valuable resource that is available on the internet as well as a smartphone application.^7^ The Wireless Information System for Emergency Responders (WISER) application provides free access to information related to hazardous materials, identification, decontamination, and health information.^8^ The WISER application also links to Chemical Hazards Emergency Medical Management (CHEMM) and the ERG Manual.^9^ The CHEMM intelligent Syndrome's tool (CHEMM-IST) can identify toxidrome and chemical substance groups in case of unknown severe exposure, while the Primary Response Incident Scene Management (PRISM) Algorithm Suggesting Proportionate Incident Response Engagement (PRISM-ASPIRE) tool aids in determining the decontamination needs of the exposed patient. There also exist Occupational Safety and Health Administration (OSHA) guidelines for hospital first receivers of victims from incidents involving hazardous substances.^1^ The National Institute for Occupational Safety and Health (NIOSH) pocketbook for chemical hazards is an important resource that will be useful in the recognition and control of occupational chemical hazards.^10^

The CHEMM website provides access to information regarding initial response, triage, decontamination, assessment, and treatment of chemically exposed patients. This website also links with two important clinical decision-making tools for substance identification and decontamination. The Radiation Emergency Medical Management (REMM) website and application provides guidelines to primary physicians about clinical diagnosis and treatment of radiation injuries during nuclear and radiological emergencies.^11^ In addition, the hospitals near any industrial area or within transportation routes should conduct a Hazard Vulnerability Analysis ( HVA) for planning purposes. The HDPRP plan should also include an Incident Action Plan (IAP) for managing HAZMAT incidents in risk areas. Healthcare workers should be trained effectively to evaluate and treat chemically exposed persons and hospitals should have a designated decontamination area in front of their emergency department. The hospital staff who are expected to work in the decontamination area must be trained at the first responders' level.^9^

## ESSENTIALS OF MANAGEMENT OF HAZMAT INCIDENT

Dedicated HAZMAT teams play a crucial role in emergency response by mitigating risks associated with chemical, biological, radiological, and nuclear hazards. These specialized teams are equipped with personal protective equipment (PPE) such as chemical-resistant suits, respirators, and gloves to ensure their safety while handling dangerous substances.^6^ Additionally, they utilize response equipment, including detection devices, decontamination units, and containment tools, to manage hazardous spills and leaks effectively.^7^ A coordinated response between local, state, and federal agencies ensures efficient hazard containment and public safety.

Effective hazardous material incident management requires comprehensive planning and training at multiple levels. Industry stakeholders develop risk management plans, while first responders follow site-specific plans aligned with State Emergency Operations Plans (EOPs) and the National Contingency Plan to ensure a structured response.^12^ Regular training and exercises help responders maintain proficiency in handling HAZMAT incidents, reinforcing best practices and ensuring preparedness for real-world scenarios.^13^ These measures collectively enhance response efficiency, minimize environmental impact, and protect communities from hazardous material threats.

At the incident site, different zones have to be established during the initial response in case of a HAZMAT incident. The

Hot Zone at the Incident site is the most hazardous area where contamination is present, and HAZMAT teams operate here with Level A PPE suits. The leak or spill of Hazardous material is located in Hot Zone. The Warm Zone is a buffer area between Hot and Cold Zone where the Decontamination is carried out. The decontamination team stay here wearing Level B PPE suits. The Cold Zone, also known as the safe zone is completely free for contamination. Medical troops, command post and staging area are located here wearing Level C or Level D PPE suits.^14^ An illustrative representation of Hot, Warm and Cold zones in Hazardous Material Incidents is given in (Figure 1).

**Figure:**
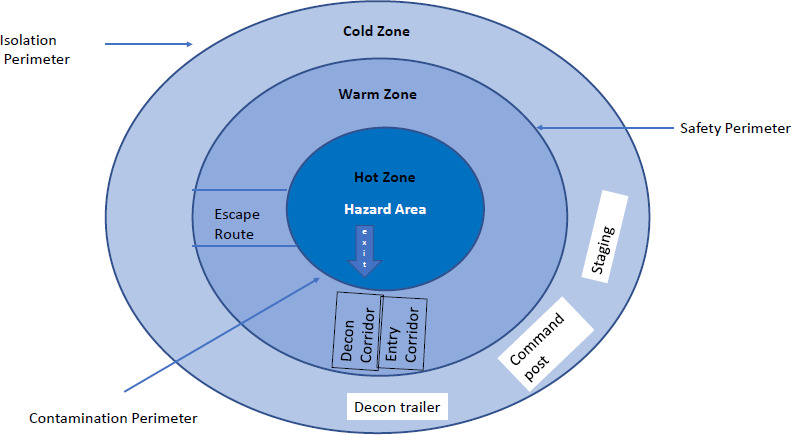


**Table 1:** HAZMAT incidents and the required level of PPE

Zone	Contamination Risk	Required PPE Level	Key Equipment
Hot Zone	High	Level A	Fully encapsulated suit, Self-containing breathing apparatus, gloves, boots
Warm Zone	Moderate	Level B	Non-encapsulating suit, Self-Containing Breathing Apparatus, gloves, boots
Cold Zone	Low Level	C/Level D	Air Purifying Respirator/Powered Air Purifying Respirator (C), Work Uniform (D)

## WAY FORWARD

Nepal urgently requires a well-structured HAZMAT Incident Management System to address industrial accidents, chemical spills, and hazardous material threats. Establishing dedicated HAZMAT response teams equipped with specialized training and modern equipment is essential for effective incident mitigation. These teams should be strategically placed in high-risk areas, such as industrial zones, major highways, and urban centers, ensuring swift emergency response. Additionally, national guidelines aligned with international standards must be developed to define operational protocols, safety measures, and inter-agency coordination.

To enhance preparedness, regular training, drills, and simulations should be conducted for first responders, including army, firefighters, police, and medical personnel. Collaboration with international organizations and neighboring countries will help strengthen Nepal's technical capacity. Moreover, integrating HAZMAT preparedness into Nepal's HDPR framework will improve coordination among emergency services, hospitals, and environmental agencies. By adopting these measures, Nepal can significantly reduce the risks associated with hazardous materials, protect public health, and ensure a safer environment.
